# Effects of radiation on the metastatic process

**DOI:** 10.1186/s10020-018-0015-8

**Published:** 2018-04-24

**Authors:** Nora Sundahl, Fréderic Duprez, Piet Ost, Wilfried De Neve, Marc Mareel

**Affiliations:** 1 Department of Radiation-Oncology, Ghent University Hospital, C. Heymanslaan 10, 9000 Ghent, Belgium; 2 Immuno-Oncology Network Ghent (ION Ghent), Ghent, Belgium

**Keywords:** Radiation, Radiotherapy, Invasion, Metastasis, Cancer

## Abstract

Radiotherapy remains one of the corner stones in the treatment of various malignancies and often leads to an improvement in overall survival. Nonetheless, pre-clinical evidence indicates that radiation can entail pro-metastatic effects via multiple pathways. Via direct actions on cancer cells and indirect actions on the tumor microenvironment, radiation has the potential to enhance epithelial-to-mesenchymal transition, invasion, migration, angiogenesis and metastasis. However, the data remains ambiguous and clinical observations that unequivocally prove these findings are lacking. In this review we discuss the pre-clinical and clinical data on the local and systemic effect of irradiation on the metastatic process with an emphasis on the molecular pathways involved.

## Background

Local invasion and distant metastasis are the cause of death in most malignant tumors. Tumor response, reflected by improved overall survival, and toxic side effects, deteriorating quality of life, receive major attention in clinical trials investigating the main treatment modalities used in cancer, namely, surgery, radiotherapy, chemotherapy, targeted therapy and immunotherapy. Yet, far less attention is paid to the effects of these treatments on tumor progression as reflected by the metastatic process.

Malignant tumors consist of cancer cells and tumor-associated host cells, both participating in invasion and distant metastasis. These cells form ecosystems at the primary and at the metastatic site, mutually communicating with one another and with stem cell-generating organs such as the bone marrow. It is highly probable that therapeutic manipulation of one ecosystem affects the others, a phenomenon that needs to be analyzed in view of the increasing cellular and molecular complexity of therapy responses (Barker et al., 2015).

Metastatic cancer cells are released from the primary tumor or from other metastases, at an undefined moment of its development, to arrive in the circulation and home at distant sites, where the ecosystem permits them to survive and either remain dormant as micro-metastases or grow to form macro-metastases (Mareel et al., 2009a). There is good evidence that cancer cells disseminate from the primary site early during tumor development (Hosseini et al., 2016), yet it is difficult to predict whether disseminated cancer cells are present at the moment of treatment and, if so, where they reside. Such cells are described as disseminated tumor cells (DTC) (Sosa et al., 2014) or sometimes as circulating tumor cells (CTC) (Kim et al., 2009). They can be awakened from dormancy by local therapeutic manipulation generating unfavorable distant effects.

Here we review the preclinical evidence on the effect of irradiation on three main steps in the metastatic process as proposed by Talmadge et al. (Talmadge & Fidler, 2010), namely angiogenesis, motility and invasion, and metastasis with an emphasis on the molecular pathways involved. Subsequently, the clinical evidence on this subject is reviewed.

## Main text

### Preclinical evidence

#### Angiogenesis

One of the first molecules implicated in enhancement of distant metastasis after irradiation of the primary tumor was angiostatin, produced by the primary tumor and keeping metastasis dormant. Elimination of the primary tumor, either by irradiation or by surgery, shifts the balance towards pro-angiogenesis and growth of the lung metastases (Table 1) (Camphausen et al., 2001). Molecular communication between ecosystems is also witnessed by the vasculogenic and pro-metastatic tumor bed effect as discussed by Kuonen et al. (Kuonen et al., 2012d) Irradiation-induced suppression of angiogenesis creates a hypoxic primary tumor ecosystem. Hypoxia stimulates hypoxia inducible factor (HIF)-dependent expression of CXCL12 and KITL promoting mobilization from the bone marrow and recruitment to primary tumor and metastatic sites of CXCR4^+^CD11b^+^ bone marrow-derived cells and KITbCD11b^+^ cells assisting vasculogenesis and metastasis respectively (Kuonen et al., 2012d). Recruitment of CD11b^+^CD11c^+^ myelomonocytic cells to the metastatic site was also found after whole thorax irradiation at a dose of 15 Gy of mice that significantly enhanced seeding and metastatic growth of intravenously injected cancer cells. Such treatment was associated with upregulation of invasion- and inflammation-promoting soluble factors, such as matrix metalloproteinase 2 (MMP2), its activator MMP14, tissue inhibitors of matrix metalloproteinase 2 (TIMP2), chemokine ligand 2 (CCL2), and urokinase-type plasminogen activator (uPA), the latter two being linked to the recruitment of the monocytic cells. Intravenous injection of multipotent vascular wall-resident mesenchymal stromal cells (MSCs) counteracted lung inflammation and metastasis by an as yet unknown mechanism (Klein et al., 2016). Translation of the latter data to the clinical situation is difficult, since whole thorax irradiation of 15 gray (Gy) is not applied in radiotherapy. Nevertheless one should consider that induction of lung metastases in murine models does occur upon total body irradiation at doses as low as 0.3 Gy (Sofia Vala et al., 2010) and upon partial thorax irradiation at doses (10 Gy) (Feys et al., 2015) that can be received by the lungs during radiotherapy for neighboring organs such as the esophagus.

**Table 1:** In vitro and in vivo experiments investigating the influence of irradiation on angiogenesis

Radiation			Vessel origin	Host	Result		Molecular	Ref
Target	Mode	Dose			Type	IR/Ctrl		
Endothelial cells								
	C-ion	0.1–8 Gy	HUVEC	Transwell chamber	Migration	< 1	αvβ3; MMP-2	(Takahashi et al., 2003)
				Collagen	Tube formation	< 1		
	kV	0.1–8 Gy		Transwell chamber		> 1		
				Collagen	Tube formation	1		
	nm	4 Gy	HUVEC	Matrigel	Tube formation	0.8	αvβ3	(Abdollahi et al., 2005)
	nm	8 Gy	HMEC	Matrigel	Invasion	1		(Kaliski et al., 2005)
	250 kV	6 Gy	HUVEC	Tissue culture plastic	Wound healing migration	4	eNOS	(Sonveaux et al., 2003)
			HUVEC	Matrigel	Tube formation			
	^137^ Cs	3 Gy	HUVEC	Matrigel	Tube formation	0.8	αvβ3	(Albert et al., 2006)
	125 kV	15 Gy	HUVEC	Tissue culture plastic	Wound healing migration	0.6		(Imaizumi et al., 2010)
	6 MV	0.5 Gy	HUVEC		Wound healing migration	> 1	VEGFR	(Sofia Vala et al., 2010)
Cancer cells								
	6MV	10 Gy	HUVEC	Transwell/CM C6	Chemotactic migration	2	MMP-2 & -9	(Parthymou et al., 2004)
	6 MV	10 Gy	CAM	CAM/C6	angiogenesis	> 1		(Parthymou et al., 2004)
			HUVEC	Matrigel/PC3	Invasion	1.9		(Abdollahi et al., 2005)
		8 Gy	HMEC	Matrigel/CM B16	Invasion	2.4	MMP-2	(Kaliski et al., 2005)
	150 kV	10 Gy	HMEC	Matrigel/CM IOMM	Tube formation	1.4	uPA	(Kargiotis et al., 2008)
	nm	8 Gy	HMEC	T. c. plastic/CM U251	Branching	1.4	MMP-2	(Badiga et al., 2011)
CAF								
	15 MV	18 Gy	HUVEC	Transwell/CM CAF	Migration	0.6		(Hellevik et al., 2013)
			HUVEC	Matrigel/CM CAF	Tube formation	1		(Hellevik et al., 2013)
Inoculation site								
	50 kV	19 Gy	CAM	CAM/C6	Angiogenesis	> 1(48 h)		(Polytarchou et al., 2004)
	6 MV	2 Gy	SCID mouse subcutis	HepG2	Micro-vessel density	> 1	VEGF	
	125 kV	20 Gy	C57/Bl subcutis	Matrigel plus FGF2	Micro-vessel density	0.36	FGF2	(Imaizumi et al., 2010)
				Matrigel plus VEGF	Micro-vessel density	0.11	VEGF	
	6 MV	0.5 Gy	nude mouse subcutis	Matrigel plus FGF2	Angiogenesis	> 1		(Sofia Vala et al., 2010)
	125 kV	20 Gy	mammary fat pad.	Mammary/AT1	Micro-vessel density	0.44		(Kuonen et al., 2012b)
Total body								
	220 kV	15 Gy	C57/Bl aortic	Tissue culture plastic	Sprouting	0.11	TGFΙβ	(Imaizumi et al., 2010)
	6 MV	0.5 Gy	zebra fish	Embryo	Angiogenesis	> 1		(Sofia Vala et al., 2010)
Tumor								
	250 kV	6 Gy	C57/Bl6 subcutis	LLC	Micro-vessel density	> 1		(Sonveaux et al., 2003)
	6MV	8 Gy	C57/BL6 cerebrum	ALTS1C1	Micro-vessel density	> 1		(Wang et al., 2013)
			SCID mouse subcutis	SW410	Micro-vessel density	2		(Timaner et al., 2015)

Abbreviations: avb3, integrin; ALTS1C1, SV40 large T-transformed astrocytes; B16, mouse melanoma cells; C57/BL, mouse strain; C6, rat glioma cells; CAF, cancer-associated fibroblasts; CAM, chick chorioallantoic membrane; C-ion, carbon ion; CM, conditioned medium; eNOS, endothelial nitric oxide synthase; FGF2, fibroblast growth factor 2; HepG2, human hepatoma cells; HMEC, human microvascular endothelial cells; HUVEC, human umbilical vein endothelial cells; IR/Ctrl, irradiated over unirradiated control target; IOMM, human meningioma cells; LLC, Lewis lung cancer cells; MMP, matrix metalloproteinase; MVD, microvessel density; nm, not mentioned; PC3, human prostate cancer cells; pcb, polycarbonate transwell chamber; SW410, human colon carcinoma cells; t.c., tissue culture; TGFb, transforming growth factor beta; U252, human glioma cells; uPA, urokinase-type plasminogen activator; VEGF, vascular endothelial growth factor; VEGFR, vascular endothelial growth factor receptor; WH, wound healing

The pro-angiogenic effect on endothelial cells by the irradiation of cancer cells was demonstrated by the subcutaneous inoculation of cancer cells, irradiated or not, in mice (Chung et al., 2006) or onto the chick chorioallontoic membrane (CAM) (Parthymou et al., 2004) with evaluation of microvessel density. Increased angiogenesis was observed even when the growth of the cancer cells was reduced after a dose of 40 Gy. In vitro coculture of endothelial cells with irradiated cancer cells in the Matrigel invasion assay without direct contact between both cell types pointed to a role for soluble mediators (Abdollahi et al., 2005). Implication of such mediators was confirmed using conditioned media from irradiated as compared to unirradiated cancer cells in assays for capillary tube formation (Kargiotis et al., 2008; Badiga et al., 2011), chemotactic migration (Parthymou et al., 2004) and Matrigel invasion (Kaliski et al., 2005). In contrast, conditioned medium from irradiated fibroblasts isolated from human lung cancer reduced transwell migration of human umbilical vein endothelial cells (HUVEC) and left tube formation on Matrigel unchanged (Hellevik et al., 2013). Irradiation of a Matrigel plug, a surrogate implant mimicking the primary tumor, 10 days after subcutaneous implantation in mice stimulated its colonization by capillaries. This finding was confirmed by the observation of enhanced infiltration of CD31-positive cells after local irradiation of Lewis lung carcinoma-bearing mice. Ex vivo irradiation-mediated enhancement of angiogenesis was demonstrated by capillary sprouting from arterioles isolated from these mouse tumors (Sonveaux et al., 2003). Also irradiation of glioma at 10 and 40 Gy on the chick CAM induces angiogenesis when administered 48 h after inoculation (Parthymou et al., 2004). After ablative doses, restoration of the vasculature in experimental glioblastoma by vasculogenesis is mediated by irradiation-induced influx of bone marrow derived monocytes and macrophages, which eventually leads to tumor regrowth (Kioi et al., 2010a; Russell & Brown, 2013a; Wang et al., 2013). Normalization of the vasculature occurred after irradiation at a dose of 5 times 5 Gy of a subcutaneous colorectal tumor in a rat where dynamic contrast-enhanced magnetic resonance imaging with gadomelitol showed reduction of neovascular leakage, enhanced tissue oxygenation and enhanced expression of vascular endothelial growth factor (VEGF) (Ceelen et al., 2006).

Irradiation of endothelial cells revealed direct anti-angiogenic as well as pro-angiogenic effects, which seems to be dependent on the irradiation dose. At doses between 2 and 15 Gy, irradiated HUVEC provoked acute (within 24 h) apoptosis and inhibited survival, proliferation, capillary tube formation on Matrigel and invasion through Matrigel (Abdollahi et al., 2005; Albert et al., 2006; Imaizumi et al., 2010). Inhibition of subsequent angiogenesis from quiescent endothelial cells about 1 week after irradiation was demonstrated in various in vivo and in vitro assays (Imaizumi et al., 2010). A lower dose (0.5 and 0.8 Gy) may exert a pro-angiogenic activity as shown by enhanced wound healing migration of HUVEC (Sofia Vala et al., 2010). Indeed, hypo-fractionated irradiation delivering more than 5 Gy per fraction destroys the microvasculature more efficiently than conventional irradiation (De Wolf et al., 2015). At biologically equivalent doses, photons stimulate migration of ECV304 human endothelial cells whereas carbon-ions work inhibitory. In line with this, capillary tube formation was abolished by carbon-ions at doses, at which it was conserved after photon irradiation (Takahashi et al., 2003).

Total body irradiation (TBI) or irradiation of host sites or sites of cancer cell implantation influenced spontaneous or cancer cell-induced angiogenesis. TBI of C57/BL mice at a high dose of 15 Gy inhibited ex vivo aortic ring formation (Imaizumi et al., 2010); at a dose smaller than 1 Gy, TBI enhanced splenocyte-induced angiogenesis in mice (Kaminski et al., 1983) and spontaneous angiogenesis in transgenic embryonic Zebra fish (Sofia Vala et al., 2010). Similarly, local irradiation was pro-angiogenic at a dose of 0.3 Gy (Sofia Vala et al., 2010) whereas higher doses, more relevant to the clinical situation, inhibited angiogenesis in the Matrigel plug assay (Imaizumi et al., 2010; Kuonen et al., 2012b). However, this inhibition of angiogenesis does not necessarily work anti-metastatic. In a model mimicking local breast cancer relapse after radiotherapy, irradiation (20 Gy) of the mouse mammary gland before injection of syngeneic AT1 cells reduced microvessel density, which was accompanied by enhanced local invasion and metastasis to lymph nodes, lungs and liver (Kuonen et al., 2012b).

Apart from the irradiation dose, the timeline also influences the angiogenic response. Implantation of C6 glioma cells on the chick CAM following irradiation entailed inhibition of vessel formation during the first hours; which was followed by attraction of new vessels within the next 48 h (Polytarchou et al., 2004).

The above summarized experiments provide arguments to accept that irradiation of cancer cells or of elements of the tumor microenvironment may support or destroy the tumor vasculature and that both support and destruction may enhance invasion and metastasis.

#### Invasion

##### Homotypic cell-cell adhesion

**Fig. 1:**
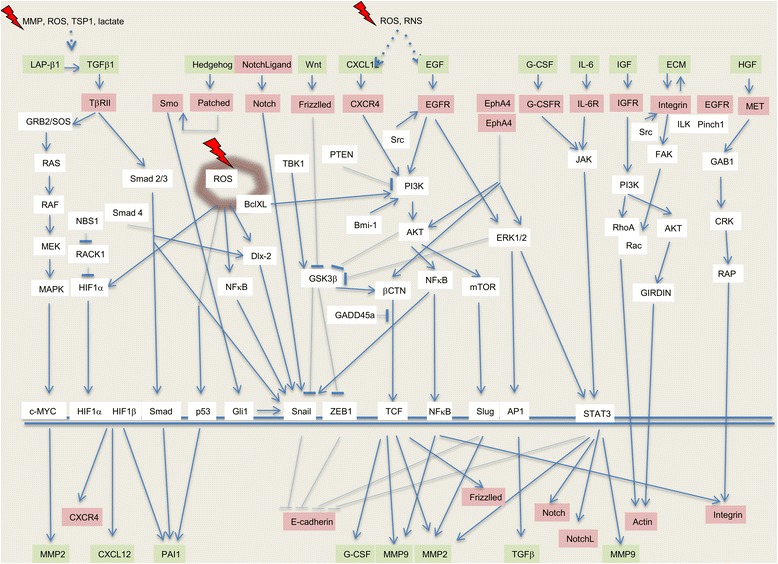
Molecular pathways regulating cellular activities implicated in radiation -enhanced invasion and metastasis. Modified after Zhai et al., (2006b), Chargari et al., (2013), He et al., (2015b) and Lee et al., (2017b), with data from: Ahmed et al., (2013b); Asuthkar et al., (2011); Bastos et al., (2014); Chang et al., 2013b; Cheng et al., (2006b); Cho et al., (2016b); Cui et al., (2015b); de Marcondes (2017); Dong et al., (2015b); Eke & Cordes, (2015b); Fujita et al., (2014b); Fujita et al., (2015c); Gu et al., (2015b); He et al., (2015b); Ho et al., (2010b); Kang et al., (2013); Yan et al., (2013b); Kim et al., (2016); Kuo et al., (2015b); Liu et al., (2014b); Park et al., (2006b); Pichard et al., (2011b); Rajput et al., (2015b); Yuan et al., (2015b); Zhai et al., (2006b); Zhou et al., (2011b). Green, red and white boxes are secretory, transmembrane receptor and intracellular signaling molecules respectively. The glowing box is a mitochondrion; the double line is DNA. Abbreviations: Akt, protein kinase B; AP1, activator protein 1; BclxL, B-cell lymphoma-extra large; Bmi-1, B cell-specific Moloney murine leukemia virus integration site 1; β-CTN, beta-catenin; CRK, CT10 regulator of kinase; CXCL12, C-X-C motif chemokine 12; CXCR4, C-X-C chemokine receptor type 4; Dlx-2, distal-less homeobox-2; ECM, extracellular matrix; EGF, epidermal growth factor; EGFR, epidermal growth factor receptor; EphA4, ephrin A 4; ERK, extracellular signal-regulated kinase; FAK, focal adhesion kinase; GAB1, GRB2-associated-binding protein; GADD45a, Growth Arrest and DNA Damage inducible Alpha; GCSF, granulocyte colony stimulating factor; GCSFR, granulocyte colony stimulating factor receptor; GIRDIN, Guanine nucleotide-binding protein α subunit -interacting vesicle-associated protein; Gli1, Glioma-associated oncogen; GRB2, Growth factor receptor-bound protein 2; GSK3β, glycogen synthase kinase 3 beta; HGF, hepatocyte growth factor; HIF-1, hypoxia-inducible factor-1; IGF, insulin-like growth factor; IGFR, insulin-like growth factor receptor; IL-6, interleukin 6; ILK, integrin-linked kinase; JAK, Janus kinase; LAP-β1, latency-associated peptide of TGF-β; MEK, mitogen-activated kinase kinase; MET, tyrosine-protein kinase Met; MMP, matrix metalloproteinase; mTOR, mechanistic target of rapamycin; NF-κB, nuclear-factor kappa-light-chain-enhancer of activated B cell; Nrf2, Nuclear factor E2 related factor 2; NSB1, Nijmegen breakage syndrome 1; p53, Tumor protein p53; PAI-1, plasminogen activator inhibitor 1; PAK1, p21- activated kinase 1; PI3K, phosphatidylinositol 3-kinase; PTEN, phosphatase and tensin homolog; Rac, Ras-related C3 botulinum toxin substrate; RACK1, receptor for activated C kinase 1; RAF, Rapid Accelerated Fibrosarcoma; RAP, Ras-proximate; RAS, Rat sarcoma; RhoA, Ras homolog gene family member A; RNS, reactive nitrogen species; ROS, reactive oxygen species; Src, sarcoma family kinase; SMAD, small and mothers against decapentaplegic; Smo, Smoothened; SOS, Son of Sevenless; STAT, signal transducer and activator of transcription; TCF, T-cell factor; TGF-β, transforming growth factor beta; TKB-1, tank-binding kinase-1; TSP1, Trombospondin 1; TβRII, TGF-β type II receptor; Wnt, Wingless-related integration site; ZEP1, Zinc finger E-box-binding homeobo× 1

The homotypic cell-cell adhesion molecule E-cadherin was identified as one of the first invasion-suppressors. Its downregulation engages epithelial mesenchymal transition (EMT) in embryonic development and in cancer (Mareel et al., 1993). Irradiation has been shown to induce EMT via the upregulation of integrin, MMP9 and MMP2 and the downregulation of E-cadherin and cytokeratin 19 (Rajput et al., 2015a), as reviewed in detail by Lee et al. (Lee et al., 2017a) Irradiation causes EMT in cancer cells from various origins, such as prostate (Chang et al., 2013a), esophagus (He et al., 2015a), lung (Ho et al., 2010a; Liu et al., 2014c; Zhou et al., 2012; Zhao et al., 2017), colorectum (Kawamoto et al., 2012; Timaner et al., 2015), breast (Rajput et al., 2015; Kim et al., 2015; Kuo et al., 2015a; Yuan et al., 2015a; Zhou et al., 2011a), uterine cervix (Yan et al., 2013a), stomach (Zhang et al., 2015b), and glioma (Park et al., 2012). This irradiation-induced EMT is also accompanied by increased cell migration and invasion (He et al., 2015a; Tsukamoto et al., 2007). Via interaction with multiple pathways, the transcription factors zinc finger protein SNAI1 (Snail) and zinc finger E-box-binding homeobo× 1 (ZEB1) appears as key in this irradiation-induced EMT (Fig. 1) (Lee et al., 2017a). Phosphorylation by kinases and dephosphorylation by phosphatases play a role in the fine tuning of irradiation-modulated EMT. The serine-threonine kinase glycogen synthase kinase-3β (GSK-3β) phosphorylates β-catenin so directing it to the ubiquitin proteasome degradation complex. Failure of GSK-3β leads to accumulation of β-catenin and translocation to the nucleus where it serves as a transcription factor in association with T-cell factor (TCF). Downstream genes of the β-catenin/TCF complex include AXIN2 (encoding axis-inhibition protein 2), FZD7 (encoding Frizzled 7), HNF1A, CCND1 (encoding cyclin D1), CD44, GCSF (encoding granulocyte-colony stimulating factor (G-CSF)), VEGF, MMP2, and MMP9, all implicated in invasion and metastasis. At the other side of the balance, the serine-threonine protein kinase tank-binding kinase-1 (TBK-1), a member of the inhibitor κB (IκB) kinase-related kinase family, activates GSK-3β, in this way attenuating irradiation-induced EMT (Liu et al., 2014c). Fractionated irradiation using sublethal doses administered over a period of 2 months and clonal selection for radioresistant esophageal cancer cells leads to downregulation of the phosphatase and tensin homolog (PTEN) and upregulation of phosphoinositide 3-kinase (PI3K) which is linked with inactivation of GSK-3β and is associated with elevated Snail protein, which enhances EMT (He et al., 2015a). Furthermore, the activation of PI3K and subsequent stabilization of β-catenin in non-small-cell lung cancer cells, via the administration of 2 Gy on 3 consecutive days, promotes G-CSF, which in turn instigates EMT through JAK-STAT signaling pathways (Cui et al., 2015a).

##### Migration

Migration of invading cells is executed through reorganization of the actin cytoskeleton and directed by the assembly/disassembly equilibrium of the cytoplasmic microtubule complex. By alteration of the biomechanical properties and the organization of the actin cytoskeleton, photon irradiation can promote invasion and migration of cancer cells, as observed in various cell lines via wound-healing assays, transwell migration assays and spheroid migration assays (see Table 2) (Kargiotis et al., 2008; Rajput et al., 2015a; Zhao et al., 2017; Kawamoto et al., 2012; Zhou et al., 2011a; Zhang et al., 2015b; Gu et al., 2015a; Tsutsumi et al., 2009; Nalla et al., 2010; De Bacco et al., 2011a; Fujita et al., 2011; Pickhard et al., 2011a; Fujita et al., 2012; Burrows et al., 2013; Ghosh et al., 2014; Murata et al., 2014; Fujita et al., 2015a; Nakayama et al., 2016; Fujita et al., 2014a; Fujita et al., 2015b; Zhai et al., 2006a; Zheng et al., 2015; Ohuchida et al., 2004). Carbon-ion irradiation can also stimulate invasion via the same PI3K pathway in certain cell lines. However, in several cell lines it was observed that carbon-ion irradiation inhibits migration and invasion via the ubiquitin-proteasome-mediated degradation of guanosine triphosphatase (GTP)-bound Rac1 and GTP-bound RhoA (Fujita et al., 2015a; Fujita et al., 2015b; Akino et al., 2009). Of note, in the majority of experiments migration and invasion were affected in the same direction by irradiation; however, an opposite effect was observed in two pancreas cancer cell lines (Qian et al., 2003) and in one breast cancer cell line (Rajput et al., 2015a) with hampered migration and improved invasion. Irradiation-enhanced invasion was also shown in organ culture where normal brain tissue fragments were confronted with aggregates of cancer cells, mimicking local invasion as it occurs in gliomas (Wick et al., 2002; Wild-Bode et al., 2001).

**Table 2:** Effect of radiation on the invasion of cancer cells in Matrigel-coated two-compartment chambers in vitro

Cancer cells		Radiation		Invasion	Molecular	Inhibitor	Ref
Origin	Code	Mode	Dose	IR/Ctrl			
Glioblastoma	U87MG; LN-18; LN-229	^137^Cs	6 Gy	5; 3; 3.7	MMP; BcL-2		(Wild-Bode et al., 2001)
	LN-229; U87MG	^137^Cs	6 Gy	1.5; 1.7	αvβ3 integrin	temozolomide	(Wick et al., 2002)
	A-172; U-138	240 kV	8 Gy	0.07; 1			(Cordes et al., 2003)
	U251; U373; LN18; LN428	^137^Cs	5 Gy	1.5; 1	EGFR/Src		(Park et al., 2006a)
	UN3; GM2	nm	6 Gy	2.2; 2.2	IGF-1/Rho		(Zhai et al., 2006a)
	U87	MV	3 Gy	1.7	Wnt/βCTN	XAV939	(Dong et al., 2015a)
	U251	200 kV	10 Gy	1.3	MMP/TIMP	Paputilone	(Furmanova-Hollenstein et al., 2013)
	C6L*	^137^Cs	3 Gy	2	EMT markers		(Park et al., 2012)
Meduloblastoma	DA0Y; D283	RS 2000	7 Gy	1.5; 1.5	uPAR; β1/FAK		(Nalla et al., 2010)
Neuroblastoma	SH-EP; SK-N-SH; SH-SY5Y; SK-N-AS; NLF	200 kV	6 Gy	4; 2.5; 4; 1; 1	HGF/Met		(Schweigerer et al., 2005)
Meningioma	IOMM-Lee	150 kV	5 Gy	1.8	uPAR		(Kargiotis et al., 2008)
Pancreatic Ca	Panc-1; Suit-2; Hs766T	^137^Cs	10 Gy	2.0; 1.6; 1	MMP-2	CGS27023A	(Qian et al., 2002)
	Panc-1; AsPC-1; Suit-2	^137^Cs	10 Gy	1.5; 1.3; 1.4	HGF/Met	NK4	(Qian et al., 2003)
	PANC-1; AsPC-1; BxPC-3; MIAPaCa-2	C-ion	2 Gy	4; 0.5; 0.3; 0.1	MMP2	GM6001	(Fujita et al., 2012)
	PANC-1	C-ion	2 Gy	2.5	NO/PI3K	Table 1	(Fujita et al., 2014a)
	AsPC-1, BxPC-3, MIAPaCa-2	C-ion	2 Gy	0.01	Rac1/RhoA		(Fujita et al., 2015a)
	MIA-PaCa; PANC-1	X-ray	4 Gy	3; 1.6	MMP-2	GM6001	(Fujita et al., 2011)
Lung SCC	EBC-1	C-ion	2 Gy	0.6			(Akino et al., 2009)
	EBC-1	4MV	2 Gy	1.2			(Akino et al., 2009)
Lung Adenoca	A549	C-ion	2 Gy	0.4	ANLN		(Akino et al., 2009)
	A459	4 MV	2 Gy	0.5			(Akino et al., 2009)
	A459	^137^Cs	10 Gy	2.5	Bcl-xl/STAT3		(Ho et al., 2010a)
	A549	C-ion	10 Gy	0.2	PI3K/Akt		(Ogata et al., 2011)
		4 Mv	10 Gy	0.5			
	A549	^60^Co	2 Gy	× 1.5	MMPs		(Zhou et al., 2012)
	A459; H1299	MV	4 Gy	0.7; 0.7	TBK1	MG132	(Liu et al., 2014c)
	A549; HT1299	^137^Cs	3 × 2 Gy	1.5; 1.5	G-CSF		(Cui et al., 2015a)
	H1299; A459; H460	MV	2 Gy	1.5; 2; 1.2	CXCR4		(Gu et al., 2015a)
	A549	^137^Cs	10 Gy	× 1.8	EGFR	Gefitinib; PPA	(Cho et al., 2016a)
	A549; H460	X-ray	4 Gy	× 1.5; × 1.4	Nrf2/Notch		(Parthymou et al., 2004; Zhao et al., 2017)
	LLC-LM*	^60^Co	7,5 Gy	× 3,5	MMP9		(Chou et al., 2012)
Mammary Ca	MCF7	^60^Co	2 Gy	× 1.4	TGF- β		(Zhou et al., 2011a)
	MCF7; SKBR3	^137^Cs	3 × 2 Gy	× 5; 4.3	SRC/PI3K		(Kim et al., 2015)
	MCF7	^137^Cs	5 Gy	× 2	NBS1/HIF-1a		(Kuo et al., 2015a)
	MCF7; MDA-MB-231		5 Gy	1.7	TGF- β	Thymoquinone	(Rajput et al., 2015a)
	MDA-MB-231		5 Gy	× 2			(Vilalta et al., 2014)
	MCF7	^137^ Cs	2 Gy	× 1.6	EMT	histamine	(Galarza et al., 2016)
	MDA-MB-231,	6 MV	10 Gy	× 2	Met	PHA665752	(De Bacco et al., 2011a)
	MCF-7	X-ray	20 × 1 Gy	× 5	Snail; twist		(Zhang et al., 2011)
	4 T1*	^137^Cs	20 Gy	× 3.5	GM-CSF		(Vilalta et al., 2014)
Prostatic Ca	PC-3; DU145	6 MV	5 × 2 Gy	×2	PI3K/AKT	BEZ235	(Chang et al., 2013)
Oral Ca	OECM1	^137^Cs	5 Gy	× 2.4			(Kuo et al., 2015a)
Esophageal. .SCC	KYSE-150	150 kV	37 Gy	× 1.3	PTEN		(He et al., 2015a)
	TE-9	125 kV	2 Gy	15	TGF- β	metformin	(Nakayama et al., 2016)
Hepatoma	HepG2; Huh7;	^60^Co	7.5 Gy	× 3	PI3K		(Cheng et al., 2006a)
Colon Ca	CaR1; DLD1	100 kV	5 Gy	6.5; 4.3	EMT		(Kawamoto et al., 2012)
	HCT116	^137^Cs	4 Gy	× 1.7	MMP		(Speake et al., 2005)
	HT29	^137^Cs	5 Gy	2	βCTN/TCF		(Bastos et al., 2014)
Cervical Ca	Siha; C33A	X-ray	75 Gy	× 3; 3	NF-kB p65		(Yan et al., 2013a)
Fibrosarcoma	HT180	proton	2 Gy	0.2	MMP-2		(Ogata et al., 2005)
		C-ion	2 Gy	0.2	MMP-2		
		4 MV	2 Gy	× 1.6 or 1	MMP-2	GM6001	
	HT1080	^137^Cs	4 Gy	× 1.5	MMP-2 & 9		(Speake et al., 2005)
	HT180	200 kV	2 Gy	× 1.5	MMP/TIMP	patupilone	(Furmanova-Hollenstein et al., 2013)
Melanoma	B16*	^137^Cs	8 Gy	× 3	MMP-2	Metastat	(Kaliski et al., 2005)

Most cell lines are human, except cell lines marked by an asterisks which are rodent. Inhibitors comprise agents that were used clinically, namely: BEZ235, an imidazoquinolone derivative and an inhibitor of PI3K and mTOR; CGS27023A, a matrix metalloproteinase inhibitor and anti-metastatic agent; Gefitinib, tyrosine kinase inhibitor targeting EGFR; GM6001, a broad -spectrum matrix metalloproteinase inhibitor known as galardin; Histamine, a biogenic amine that targets four (H1–H4) histamine subtypes G-protein coupled receptors (GPCR); Metastat, a chemically modified tetracycline and inhibitor of gelatinase and MMP; Metformin, an anti-diabetic; MG132, a specific proteasome inhibitor; NK4, a four-kringle antagonist of hepatocyte growth factor and an angiogenesis inhibitor; Patupilone, a microtubule stabilizing drug; PHA665752, an ATP-competitive small-molecule inhibitor of the tyrosine kinase c-Met; PPA, Podophyllotoxin acetate, a microtubule inhibitor and a spindle poison; temozolomide, an alkylating chemotherapeutic drug; Thymoquinone, a regulator of NF- kB and extracellular signal-regulated kinase (ERK) signaling pathways. Abbreviations: Akt, protein kinase B; ANLN, human homologue of anailin; b1, beta1 integrin subunit, b-CTN, beta-catenin; BcL-2, B-cell lymphoma 2; BclxL, B-cell lymphoma-extra large; C-ion, carbon-ions; Ca, carcinoma; Co, cobalt; Cs, cesium; Ctrl, unirradiated control; CXCR4, C-X-C chemokine receptor type 4; EGFR, epidermal growth factor receptor; EMT, epithelial-to-mesenchymal transition; FAK, focal adhesion kinase; GCSF, granulocyte colony stimulating factor; Gy, gray; HGF, hepatocyte growth factor; IGF1, insulin-like growth factor-1; Met, tyrosine-protein kinase Met; MMP, matrix metalloproteinases; nm, not mentioned; NO, nitric oxide; Nrf2, Nuclear factor E2 related factor 2; PI3K, phosphatidylinositol 3-kinase; PPA, Podophyllotoxin acetate; Rho, Ras homolog gene family member; SCC: squamous cell carcinoma; Src,sarcoma family kinase; STAT, signal transducer and activator of transcription; TBK1, tank-binding kinase 1; TCF, T-cell factor; TGFb, transforming growth factor beta; TIMP, tissue inhibitor of matrix metalloproteinase; TGF-b, transforming growth factor beta; uPAR, urokinase-type plasminogen activator receptor; Wnt, Wingless-related integration site; XAV 939, a selective β-catenin-mediated transcription inhibitor

Various elements of the ecosystem served as successful targets for irradiation-mediated enhancement of invasion. Cancer cells, mostly issued from cell lines, were target in most studies (Table 2); host cells were targets in some experiments. For instance, irradiation of reconstituted vascular wall led to increased degradation by unirradiated HT1080 sarcoma cells (Heisel et al., 1984). Irradiated EA.hy926 endothelial cells enhanced the migration of human MDA-MB-231 and murine 4 T1 breast cancer cells (Feys et al., 2015). Enhanced colon cancer cell adhesion to HUVEC was obtained when both elements were irradiated (Hamalukic et al., 2011a). Variations in the elements of the ecosystems and of the experimental manipulations influenced irradiation-enhancement of invasion-related cellular activities, though various authors did not always obtain the same result.

The invasive response to irradiation seems to be dose-dependent. Dose-response curves were registered for single doses between 1 Gy and 10 Gy (Burrows et al., 2013; Park et al., 2006a). Maximal enhancement of invasion-related activities was obtained at single doses around 4 to 5 Gy (Park et al., 2012; Fujita et al., 2011). In most of these experiments with sublethal doses, proliferation was inhibited, a situation that is known to be permissive for migration and invasion (Storme & Mareel, 1984). Proliferation was unchanged in human glioma cells at the lower dose of 20 cGy that led to enhanced wound-healing migration (Ghosh et al., 2014). To obtain more invasive radioresistant cell lines, doses varied largely: 1 × 6 Gy (Su et al., 2012); 1 × 10 Gy (Tsutsumi et al., 2009); 5 × 2 Gy daily (Chang et al., 2013); 20 × 1 Gy (Zhang et al., 2011) and 35 × 2 Gy (He et al., 2015a; Yan et al., 2013a). In the experiments by Zhang et al. cells were tested shortly after irradiation, in contrast to the others where irradiation occurred weeks before testing in the in vitro ecosystem as they were maintained and selected to obtain resistant cell lines (Zhang et al., 2011). The pro-invasive effect of 10 Gy on lung adenocarcinoma cells was transient with enhancement after 16 and 24 h but not after 48 and 72 h (Ho et al., 2010a).

As to the organ of origin, Fujita et al. compared 31 cell lines irradiated or not with a single dose and found qualitative (e.g. stimulation of inhibition of invasion) and quantitative (e.g. the extent of stimulation) differences in their invasive response, that were not related to their organ of origin (Fujita et al., 2015b). In the same experimental series, differences in invasive response to irradiation were found between cancer cells of similar organ of origin, namely glioma (Cordes et al., 2003), lung (Akino et al., 2009), thyroid (Burrows et al., 2013), and breast cancer (Paquette et al., 2011). Opposite effects of irradiation were described for the same cell lines also by different authors, e.g. MCF7 breast cancer cells (Kim et al., 2015; Paquette et al., 2011; Galarza et al., 2016), pancreatic cancer cells (Fujita et al., 2012; Qian et al., 2002), glioblastoma cells (Wick et al., 2002; Wild-Bode et al., 2001; Cordes et al., 2003) and lung cancer cells (Zhao et al., 2017; Zhou et al., 2011a; Gu et al., 2015a; Jung et al., 2007; Cho et al., 2016a; Ogata et al., 2011; Liu et al., 2014a). Published details of these experiments did not permit us to conclude which variables were responsible for such opposite results. With regard to the mode of irradiation, no systematic differences were noted between photons with lower (250 kV) as compared to higher (MV) energy (Table 2). Heavy particles (carbon-ions) were clearly different in as much as they inhibited invasion at biologically equivalent or lower doses that lead to enhancement with photons (Fujita et al., 2012; Fujita et al., 2015a; Fujita et al., 2015b). This was also demonstrated in matched experiments using the same cell types and the same cellular activities as an endpoint (Ogata et al., 2005; Rieken et al., 2012; Stahler et al., 2013), though there are exceptions that showed stimulation (Murata et al., 2014) or inhibition (Akino et al., 2009; Ogata et al., 2011) for both modes of irradiation.

Epidermal growth factor receptor (EGFR)-positive breast cancer cells, but not negative ones, were induced to invade by ionizing radiation in a reactive oxygen species (ROS)-dependent manner (Kambach et al., 2014). In human lung adenocarcinoma cells (A549), ROS function as intracellular second messengers that participate in the activation of intracellular signaling pathways and, therefore, may mediate ligand-independent and rapid (within 5 min) phosphorylation of cell surface receptors, like EGFR (Lee et al., 2008). Kinetic analysis showed that the non-receptor kinase Src was transiently activated within 30 min of irradiation at a dose of 10 Gy, whereas ErbB2 phosphorylation began to increase after 60 min and was maintained for at least 24 h. The signal transducer and activator of transcription factor 3 (STAT3) was observed to be phosphorylated also 30 min after irradiation, preceding the accumulation of the Bcl-2 anti-apoptotic family member B cell lymphoma-extra large (Bcl-X_L)_ and stimulation of invasion (Table 2) (Ho et al., 2010a). In the latter experiments, applying a single exposure of 10 Gy, irradiation-induced STAT3 phosphorylation was not stable, and decreased with continued incubation, reflecting a downregulation of malignant signals. Indeed, Bcl-X_L_ and EMT markers were restored to their original levels when incubation continued for up to 48–72 h and invasion did no longer differ from that of unirradiated controls. It would be interesting to know whether or not this reversibility could be overcome by a multiple exposures as it is applied in fractionated radiotherapy. Curiously and in line with the results on invasion, carbon-ions do not affect EGFR activity or the downstream signaling effectors (Fujita et al., 2015b; Stahler et al., 2013).

##### Cell-matrix adhesion

Cell-matrix adhesion is mediated by transmembrane receptors, called integrins that bind to the extracellular matrix and signal intracellularly. Integrins contribute to invasion and metastasis by regulating cell motility, localization and activity of MMPs, as well as cell survival (Desgrosellier & Cheresh, 2010). Integrins are linked to the EGFR through integrin-linked kinase (ILK) and particularly interesting new cysteine-histidine-rich 1 (PINCH1) forming multiprotein and multifunctional complexes cooperatively promoting invasion (Li et al., 2013; Eke & Cordes, 2015a). At their extracellular end αvβ5 integrins cooperate with the matricellular protein cysteine-rich angiogenic inducer 61 (CYR61) mediating the pro-metastatic effect of tumor bed irradiation (Monnier et al., 2008). In vitro and in vivo experiments (Tables 2 and 3) show that invasion-enhancing doses of irradiation upregulate integrins as well as phosphorylate focal adhesion kinase (FAK) and paxillin, which are critical mediators of cell migration (Tsutsumi et al., 2009; Nalla et al., 2010; Gogineni et al., 2011). In pancreatic cancer cells upregulation of integrins was ascribed to increased transcription and to increased postendocytic recycling (Yao et al., 2011). A striking correlation between glioma cell migration and upregulation of αvβ5 integrin is described by Rieken et al. (Rieken et al., 2011) In contrast to photons, carbon-ions impaired migration through down-regulation of cell surface integrin expression. Also MMPs are activated upon irradiation-enhanced integrin expression, suggesting an interplay between the mesenchymal form of migration and cell matrix degradation (Wild-Bode et al., 2001; Yao et al., 2011; Vehlow & Cordes, 1836). A forward feedback survival pathway was discovered in radioresistant breast cancer cells, where irradiation-stimulated β1 integrin causes NF-κB to bind to the promoter region of β1 integrin and to transactivate it (Ahmed et al., 2013a).

**Table 3:** Effect of radiation on local invasion or metastasis of rodent and human cancer cell lines as tested in syngeneic and in xenogeneic mice respectively

Radiation			Inoculum			Score (Ir/Ctrl)	Molecular	Ref
Target	Mode	Dose	Code	Origin	Site			
				Syngeneic				
Cancer cells								
	^137^ Cs	3 Gy	9 L	glioma	intracaranial	40^d^	αvβ3	(Wild-Bode et al., 2001)
	C-ion	5 Gy	LM8	osteosarcoma	subcutaneous	0.6		(Ogata et al., 2005)
	C-ion	5 Gy	LM8	osteosarcoma	intravenous	0.4		(Ogata et al., 2005)
	4 MV	10 Gy	LM8	osteosarcoma	subcutaneous	1		(Ogata et al., 2005)
	4 MV	10 Gy	LM8	osteosarcoma	intravenous	0.4		(Ogata et al., 2005)
	nm	3 Gy	9 L	glioma	intracaranial	2.4^d^	IGFR1	(Zhai et al., 2006a)
CAF								
	^137^ Cs	10 Gy	Suit_2/CAF	pancreatic ca	intrapancreas	2.5^d^	Met	(Ohuchida et al., 2004)
	nm	4 Gy	PC/CAFCM	Pancreatic ca	intravenous	4	CXCL12	(Li et al., 2016a)
Mammary f. p.								
	MV	4 × 6 Gy	D2A1/FUCCI	mammary ca	mammary f.p.	1.8^d^	COX2; IL6	(Bouchard et al., 2013)
	MV	4 × 6 Gy	D2A1/FUCCI	mammary ca	mammary f.p.	2^c^		(Bouchard et al., 2013)
	MV	4 × 6 Gy	D2A1/FUCCI	mammary ca	mammary f.p.	2.4		(Bouchard et al., 2013)
	MV	4 × 6 Gy	D2A1/FUCCI	mammary ca	intravenous	1		(Bouchard et al., 2013)
	125 kV	20 Gy	AT1	mammary ca	mammary f.p.	6^d^	HIF/KITL	(Kuonen et al., 2012b)
	125 kV	20 Gy	AT1	mammary ca	mammary f.p.	8^b^		
	125 kV	20 Gy	AT1	mammary ca	mammary f.p.	6		
Thorax								
	300 kV	10 Gy	MMT/PyVmT	mammary ca	intravenous	4	TGFβ	(Biswas et al., 2007)
	300 kV	10 Gy	MMT/PyVmT	mammary ca	mammary f.p.	17^c^	TGFβ	(Biswas et al., 2007)
	300 kV	10 Gy	MMT/PyVmT	mammary ca	mammary f.p.	17	TGFβ	(Biswas et al., 2007)
	^60^ Co	1 × 15 Gy	TS/A	mammary ca	intravenous	2.5		(Klein et al., 2016)
	^60^ Co	1 X15 Gy	B16/F10	melanoma	intravenous	2.3	uPA;CCL2	
Partial lung								
	220 kV	10 Gy	4 T1	mammary ca	mammary f.p.	3		(Feys et al., 2015)
Abdomen								
	6 MeV	2 Gy	CT26	colon ca	intravenous	> 1		(Timaner et al., 2015)
Total Body								
	^60^ Co	10 Gy	B16/F10	melanoma	subcutaneous	2		(Klein et al., 2016)
Tumor								
	250 kV	10 × 0.6 Gy	KLN-205	sarcoma	subcutaneous	1^a^		(Baker et al., 1981)
	^137^ Cs	5 × 10 Gy	LLC-LM	lung ca	subcutaneous	10		(Camphausen et al., 2001)
	^137^ Cs	5 × 10 Gy	T241	fibrosarcoma	subcutaneous	14	angiosatin	(Camphausen et al., 2001)
	6 MV	8 Gy	ALT-S1C1	glioma	intracranial	0.7		(Wang et al., 2013)
	^60^Co	5 × 10 Gy	LLC-M	lung ca	subcutaneous	5	MMP9	(Chou et al., 2012)
				Xenogeneic				
Cancer cells								
	^60^Co	7.5 Gy	HepG2	Hepatoma	intrahepatic	3^d^	MMP9	(Cheng et al., 2006a)
	^60^Co	4 Gy	CHO1	Fibrosarcoma	intravenous	5		(Hamalukic et al., 2011a)
	MV	6 Gy	CaSki	Cervical ca	intravenous	1.6	Ras	(Su et al., 2012)
	MV	2 Gy	HT1299	Lung ca	intravenous	6a	CXCR4	(Gu et al., 2015a)
Inoculation site								
	220 kV	20 Gy	R18	Melanoma	intradermal	2^b^	O_2_/uPAR	(Rofstad et al., 2005)
	220 kV	20 Gy	D12	Melanoma	intradermal	2	O_2_/IL8	(Rofstad et al., 2005)
	220 kV	20 Gy	SCCVII	Oral ca	subcutaneous	3.5	CYR61; αvβ5	(Monnier et al., 2008)
	220 kV	20 Gy	HCT116	Colon ca	subcutaneous	4		
Total body								
	6 MV	0.3 Gy	4 T1	mammary ca	mammary f.p.	7	VEGFR	(Sofia Vala et al., 2010)
	6 MV	0.3 Gy	MOLT4	leukemia	intravenous	5		
Tumor								
	220 kV	10 Gy	R18	Melanoma	intradermal	1.6^b^	O_2_/uPAR	(Rofstad et al., 2004)
	250 kV	2 × 4 Gy	U251	glioma	intracranial	> 1^d^	MMP2	(Badiga et al., 2011)
	^60^Co	5 × 10 Gy	C6L	glioma	subcutaneous	> 1^a^	EMT	(Park et al., 2012)
	nm	5 × 2 Gy	FCT133	Thyroid ca	subcutaneous	1.6	PI3K	(Burrows et al., 2013)
	nm	20 Gy	4 T1	mammary ca	mammary f.p.	5^e^		(Vilalta et al., 2014)
	6 MeV	2 Gy	SW480	Colon ca	intracolonic	> 1^a^		(Timaner et al., 2015)
	X-rays	15 × 2 Gy	SGC7901	Gastric ca	subcutaneous	2		(Zhang et al., 2015a)

Scores indicate the metastasis in the lungs unless indicated as: ^a^, lung and other metastases; ^b^, lymph node metastasis; ^c^, circulating tumor cells (CTC); ^d^, Local invasion; ^e^, self-seeding from an unirradiated heterolateral breast tumor to an irradiated recipient one. Scores represent metastasis after administration of radiation relative to control mice (Ir/Ctrl). Abbreviations; avb3, integrin; CAF, cancer-associated fibroblasts; C-ion, carbon-ions; Ca, carcinoma; CAF, cancer-associated fibroblasts; CCL2, C-C motif chemokine ligand 2; CM, conditioned medium; Co, cobalt; COX-2, cyclooxygenase-2; Cs, cesium; CTC, circulating tumor cells; CXCL12, C-X-C motif chemokine 12; CXCR4, C-X-C chemokine receptor type 4; CYR61, Cysteine-rich angiogenic inducer 61; EMT, epithelial-to-mesenchymal transition; f.p., fat pad; Gy, gray; HIF, hypoxia-inducible factor; IGFR, insulin-like growth factor receptor; IL, interleukin; KITL, tyrosine-kinase receptor encoded by the KIT locus, also known as stem cell factor; Met, tyrosine-protein kinase Met; MMP, matrix metalloproteinases; nm, not mentioned; PI3K, phosphatidylinositol 3-kinase; Ras, small GTPase encoded by the RAS gene; sa, sarcoma; TGF-b, transforming growth factor beta; uPAR, urokinase-type plasminogen activator receptor; VEGFR, vascular endothelial growth receptor

##### Proteolysis

Breakdown of extracellular matrix (ECM) by invasive cells is mediated by lytic enzymes such as MMP2, MMP9 and membrane-bound MMP-1 (MT1-MMP). These enzymes act in balance with inhibitors such as TIMPs. Proteases not only degrade ECM but also cleave off growth factors from their precursors, e.g. proteases activate TGF-β through cleavage from its inactive precursor latency-associated peptide (LAP-β1). Both enzymatic activities are enhanced by ionizing radiation, explaining stimulation of invasion and metastasis-related cellular activities (Tables 2 and 3). Irradiation enhanced activity of MMP9 is explained by enhanced transcription via NF-κB (Park et al., 2006a; Cheng et al., 2006a). Irradiation-enhanced MMP2 promoter activity, mRNA transcription, and protein secretion associated with invasiveness was observed in glioma cells lacking functional PTEN (U87, U251, U373, and C6) but not in those harboring wild-type PTEN (LN18 and LN428) (Park et al., 2006a; Cordes et al., 2003). This is in contrast with the observations of Wild-Bode et al. who reported upregulated expression of MMP2 and MT1-MMP, downregulated expression of TIMP-2, and increased invasiveness in glioma cells regardless of PTEN status (Wild-Bode et al., 2001). In LLC-LM murine lung cancer cells, irradiation prompted increased expression of mRNA and protein as well as increased enzyme activity for MMP9 but not for MMP2 (Chou et al., 2012). These researchers also observed that the administration of 5 × 10 Gy to the subcutaneous tumor in the thigh enhanced the MMP9 level, stimulated entry of cancer cells into the circulation and increased the number of pulmonary metastasis, whereas 2 × 30 Gy reduced these parameters. Implication of the β-catenin/TCF signaling pathway links MMP to the EMT phenotype. In U87 glioma cells, irradiation increased the β-catenin/TCF transcriptional activity, followed by an upregulation of its downstream genes, MMP2, and MMP9 (Dong et al., 2015a). Interestingly, irradiation-induced overexpression of growth arrest and DNA damage inducible protein alpha (Gadd45a) inhibits the nuclear translocation of β-catenin, resulting in a downregulation of MMP9, an increase in E-cadherin and a delayed medulloblastoma tumor development (Asuthkar et al., 2011). Other hydrolases are also involved in irradiation-induced invasion, e.g. the activation of the serine protease uPA in pancreatic cancer cells via carbon-ion irradiation (Fujita et al., 2014a).

As stated above, activation of TGF-β through release from LAP-β1 is an extracellular process, that occurs rapidly (within 24 h), persistently (7 days) and dose-dependently (from 0.1 Gy to 5 Gy) upon irradiation of the mouse mammary gland (Ehrhart et al., 1997). Such activation results from ROS-induced oxidation of LAP-β1 triggering a conformational change that releases TGF-β1. A methionine residue at amino acid position 253 unique to LAP-β1 is critical to this ROS-mediated activation (Jobling et al., 2006). Circulating levels of TGF-β were increased in a mouse model of irradiation-enhanced metastatic breast cancer, regardless of the site of irradiation: thorax, pelvis or primary tumor and before or after injection of the cancer cells. In this experiment, circulating cancer cells did not grow ex vivo in the presence of the TGF-β antibody and irradiation failed to enhance lung metastases in mice bearing tumors that lacked the type II TGF-β receptor, suggesting that the increase in metastases was due to a direct effect of TGF-β on the cancer cells (Biswas et al., 2007).

##### Heterotypic cell-cell adhesion

Heterotypic cell-cell adhesion, implicated in extravasation and metastasis, is facilitated by irradiation-triggered expression of cell adhesion factors such as E-selection and vascular cell adhesion protein (VCAM) on cancer cells and endothelial cells (Hamalukic et al., 2011). Also cell-substrate adhesion is enhanced by irradiation as quantified by the number of cells that resist washing after seeding and incubation on tissue culture plastic substrate coated with elements from the extracellular matrix (Tsutsumi et al., 2009; Nalla et al., 2010; Akino et al., 2009; Cordes et al., 2003; Jung et al., 2007; Ogata et al., 2005).

The type of extracellular matrix influenced clonogenic survival of some glioblastoma cells with irradiation-enhancement on fibronectin or Matrigel, but not bovine serum albumin (Cordes et al., 2003). For irradiation-enhanced cell-substrate adhesion, little or no difference was found between collagen 1, fibronectin, vitronectin or laminin (Akino et al., 2009; Ogata et al., 2005; Lee et al., 2010). Irradiation-enhanced cell-substrate adhesion and wound-healing migration could be obtained only when the tissue culture substrate was coated with collagen 1 (Tsutsumi et al., 2009).

The pro-invasive effect of fibroblasts on pancreatic cancer cells has been ascribed to hepatocyte growth factor (HGF) secreted by the fibroblasts and interacting with the tyrosine-protein kinase Met (c-MET) on the cancer cells by Ohuchida et al. (Ohuchida et al., 2004), and to the ubiquitous and polyfunctional chemokine CXCL12 interacting with CXCR4 on the cancer cells by Li et al. (Li et al., 2016a) HGF was put forward because of the activation of c-MET in pancreatic cancer cells exposed to irradiated fibroblasts and neutralization of this effect by NK4 (an antagonist of HGF), although increased release of HGF from the fibroblasts could not be demonstrated. CXCL12 was selected because it emerged as the most elevated species among the irradiation-enhanced levels of chemokine mRNA. To confirm, Li et al. showed that addition of CXCL12 to the co-culture stimulated invasion, whereas the CXCR4 partial agonist AMD3100 reduced it. CXCL12 together with macrophage migration inhibitory factor (MIF) were chosen also to explain stimulation of adhesion and trans-endothelial migration of human breast cancer cells by irradiation of lung cells on the basis of a high fold change found in a 52 cytokine secretome assay (Li et al., 2016a). These pro-invasive cellular activities were largely mimicked by recombinant CXCL12 and MIF, whereas an allosteric inhibitor of the CXCR4 receptor prevented these activities (Feys et al., 2015).

In summary, irradiation might stimulate motility and invasion on various different levels. This effect seems to be dependent on dose, cell line and type of irradiation (photons versus heavy ions), and was sometimes only transient.

#### Metastasis

To analyze the mechanisms of metastasis, cancer cells were inoculated at various sites, orthotopic or paratopic, in immunosuppressed or syngeneic hosts (Table 3), producing models that are more readable than the natural situation at the risk of being less relevant. Similarly, manipulation of the target and the time of radiation facilitates dissection of mechanism at the price of lower relevance. In this way an increase in the metastatic potential has been observed upon radiation of the cancer cells or of the host, be it on the site of inoculation, putative sites of metastasis or whole body, prior to transfer of cancer cells. Also the intravenous inoculation of radio-resistant cancer cells that were sub-cultured for several passages after irradiation yielded an increased metastatic potential (Su et al., 2012). The transfer of cancer cells into preirradiated host tissue is a model of recurrent primary tumors in humans. This model has proven the tumor bed effect, a phenomenon which was originally described as a prolonged latency period and a reduced volumetric growth rate of tumors implanted in previously irradiated tissue and was later extended to an increased metastatic potential of tumors in preirradiated beds (Stenstrom et al., 1955; Milas et al., 1988; Rofstad et al., 2005). For instance, irradiation of the intradermal inoculation site in nude mice caused enhanced lung metastasis from human melanoma tumors (Rofstad et al., 2005) and irradiation of the mammary fat pad of syngeneic mice 24 h before inoculation of D2A1 breast cancer cells enhanced invasion, CTCs and lung metastasis upon orthotopic but not upon intravenous inoculation (Bouchard et al., 2013).

As for irradiation of putative sites of metastasis, contradictory results have been noted. On the one hand, Travis et al. observed that irradiation of the whole lung (14,5 or 20 Gy) after the subcutaneous growth of sarcoma F tumors in CBA mice led to a decrease in lung metastases (Travis et al., 1981). On the other hand, several others have reported an increase in lung metastases after irradiation of the lungs prior to subcutaneous injection of TS/adenocarcinoma cells or B16 melanoma cells or prior to intravenous injection of B16 melanoma cells, TS/adenocarcinoma cells (Klein et al., 2016), fibrosarcoma cells (Withers & Milas, 1973), mammary carcinoma cells (Dao & Yogo, 1967), Ehrlich ascites cells (Tanaka, 1976) or Walker tumor cells (Fisher & Fisher, 1969). Furthermore, Gong et al. reported that in BALB/c mice, irradiation of the right side of the chest to 9 Gy after injection of 4T1 mammary carcinoma tumor cells in the right last mammary gland led to higher incidence of lung metastasis and a shorter survival (Gong et al., 2015). Interestingly, Feys et al. observed enhanced lung metastasis from orthotopic 4T1mouse mammary tumors after partial right lung irradiation, but not after whole thorax irradiation (Feys et al., 2015). Also irradiation of the liver has been reported to lead to an increase in liver metastases after the intraportal inoculation of Ehrlich ascites carcinoma cells (Fisher & Fisher, 1969; Koike et al., 1962). Several hypotheses of this pro-metastatic effect of radiotherapy on the putative site of metastasis have been formulated. (a) Circulating tumor cells might be attracted to the irradiated tissue. (b) Cancer cell release from the primary tumor might be stimulated. (c) Irradiation could instigate local immune changes leading to the recruitment of several immune cells from the innate and adaptive immune system, which can produce paracrine signals facilitating tumor survival and growth (Feys et al., 2015; Gong et al., 2015). These immune cells can however also stimulate an antitumor immune response, which is therefore not in line with the previously discussed observations. (d) Irradiation could damage vascular endothelial cells which enables the transmigration of tumor cells through the endothelium (Klein et al., 2016).

Via bioluminescence it was proven that irradiation of the primary breast tumor increases the homing of donor tumor cells to this site and therefore enhances self-seeding (Vilalta et al., 2014). Timaner et al. showed in mice that not only local irradiation of the tumor with 2 Gy will cause an increase in metastases, but also that irradiation (2 Gy) of the abdomen, prior to intravenous injection of CT26 colon cancer cells into the tail vein leads to higher mortality due to pulmonary metastases (Timaner et al., 2015). This finding indicates that irradiation can instigate a systemic host response that enhances migration and invasion of tumor cells.

Delivery of an ablative dose to the primary tumor and a subsequent increase in lung metastases as compared to no irradiation of the primary tumor led Camphausen et al. to conclude that metastases are present in a dormant form at the moment of irradiation, similar to the experiments in which the same type of tumors were removed surgically (Camphausen et al., 2001; O'Reilly et al., 1994). With a subtherapeutic dose, irradiation-enhanced metastasis was observed only after regrowth of the primary tumor, leading to the conclusions that irradiation stimulated metastasis from the primary tumor (Rofstad et al., 2005).

**Fig. 2:**
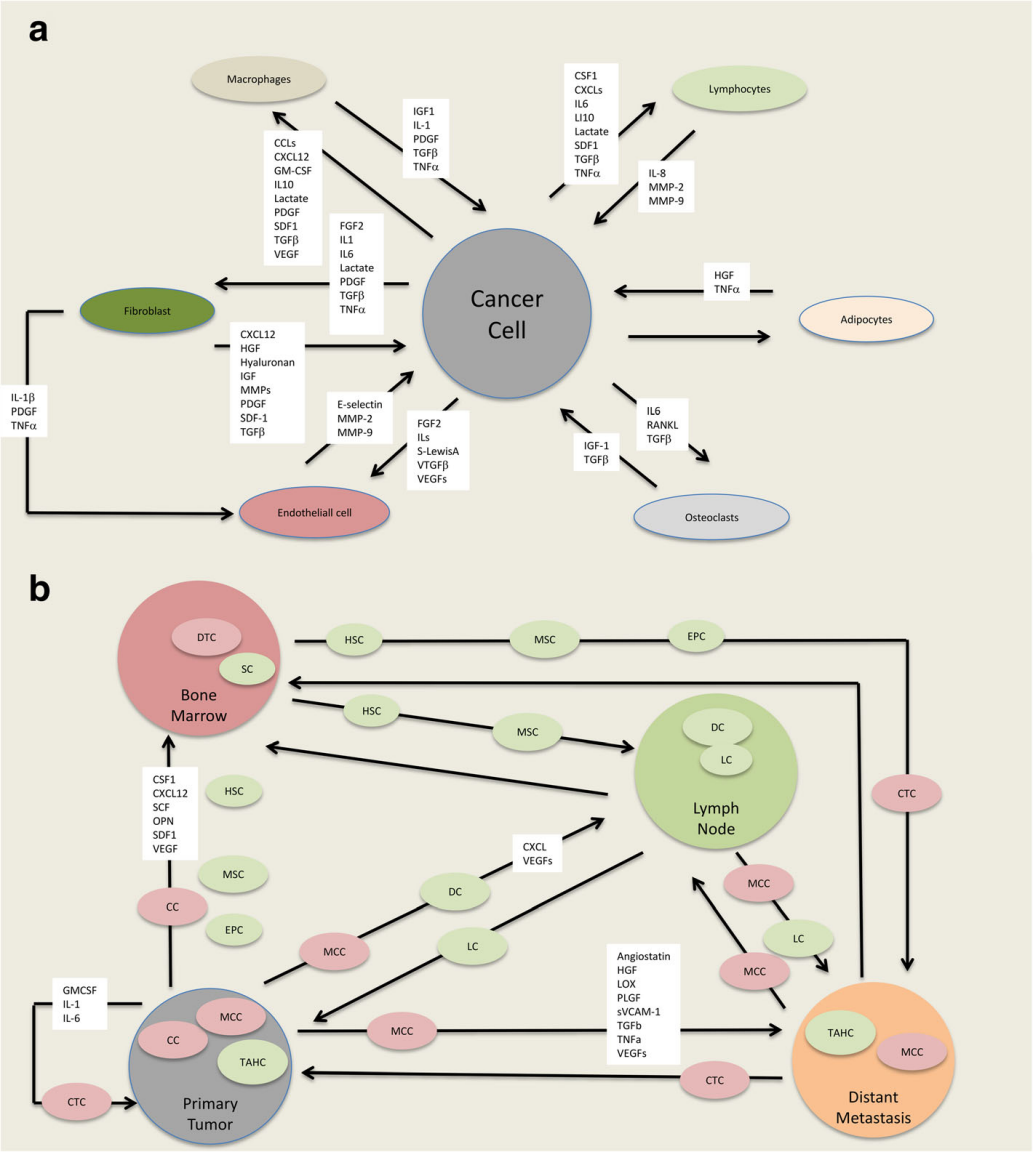
Schematics of mutual communication between cancer cells, tumor associated host cells and ecosystems. **a** Schematic of communication between cancer cells and tumor-associated host cells with invasion-related radiation-sensitive molecules. Arrows indicate the communication between cancer cells and tumor-associated host cells, namely fibroblasts, macrophages, lymphocytes, adipocytes, osteoclasts and endothelial cells, and between tumor-associated host cells, for example fibroblasts to endothelial cells. White text boxes overlapping the arrows contain the molecules implicated in this communication and shown to be sensitive to alterations by radiation. This intercellular communication establishes the individual local ecosystems that participate at metastasis as shown in panel **b** Adapted from Mareel et al., (2009b), with data from: Vakaet (2004); Abdollahi (2005); Chargari et al., (2013); De Bacco et al., (2011b); Gu et al., (2015b); Hamalukic et al., (2011b); Hirschhaeuser et al., (2011); Kuonen et al., (2012a); Li et al., (2016b); Madani et al., (2008); Nubel (2004); Vilalta (2016); Lee et al., (2017c). **b** Schematic of the communication between ecosystems of primary tumor, distant metastasis, lymph node and bone marrow, associated with metastasis and sensitive to ionizing radiation. Arrows indicate the communication between ecosystems, namely the primary tumor, lymph node, distant metastasis and bone marrow. Participating at this communication are host cells (smaller green ovals), cancer cells (smaller pink ovals) and molecules (white text boxes), all implicated in radiation-enhanced metastasis. Adapted from: Madani et al., (2008); Ceelen et al., (2014) and Willaert et al., (2014) with data from: Kioi et al., (2010b); Kuonen et al., (2012c); Russell & Brown (2013b); Vilalta (2016). Abbreviations: CC, cancer cells; CCL, C-C motif chemokine ligand; CSF1, macrophage colony-stimulating factor 1; CTC, circulating tumor cells; CXCL, C-X-C motif chemokine ligand; DC, dendritic cells; DTC,disseminated tumor cells; EPC, endothelial precursor cells; FGF2, basic fibroblast growth factor; GMCSF, granulocyte-macrophage colony-stimulating factor; HGF, hepatocyte growth factor; HSC, hematopoietic stem cells; IGF1, insulin-like growth factor 1; IL, interleukin; LC, lymphocytes; MCC, metastatic cancer cells; MMP, matrix metalloproteinase; MSC, mesenchymal stem cells; OPN, osteoprotogerin; PDGF, platelet-derived growth factor; PlGF, placental growth factor; RANKL, receptor activator of nuclear kappa-B ligand; SC, stem cells; S-Lewis A, sialyl-Lewis A antigen; SCF, stem cell factor; SDF1 (also called CXCL12), stromal- cell derived factor 1; sVCAM1, soluble vascular cell adhesion molecule 1; TGFβ, transforming growth factor beta; TNFα, tumor necrosis factor-alpha; VEGF, vascular endothelial growth factor

An overview of the communication within the cancer ecosystem regarding invasion and metastasis is depicted in Fig. 2. The molecular pathways implicated in the process of radiation-enhanced invasion and metastasis is depicted in Fig. 1. There is large and convincing preclinical evidence in favor of a prometastatic activity of radiation with very few scores lower than zero (seventh column in Table 3), pointing to inhibition. Furthermore, in vitro and in vivo experiments have revealed a profusion of molecular pathways implicated in radiation-enhanced invasion and metastasis (Fig. 1), several of which are sensitive to inhibition by molecules that are clinically applicable (Table 2). The crucial question is: how do these experimental findings translate to the clinical situation?

### Clinical evidence

Preclinical evidence on how irradiation influences the metastatic process is conflicting, and in vitro and in vivo experiments are often not a good representation of the human situation. Furthermore, the potential pro-metastatic effect of radiotherapy is hard to infer from the literature due to lack of attention to metastasis in palliative cases, heterogeneity of the cohorts analyzed, lower sensitivity or specificity of methods of detection, lack of systematic autopsy and weak study designs. Nonetheless, clinical evidence exists that certain cancer therapies produce pro-metastatic and pro-invasive signals.

Chung et al. analyzed a cohort of 340 patients with unresectable hepatocellular carcinoma (T3N0M0) who received either transcatheter arterial chemoembolization alone or in combination with radiotherapy (40–66 Gy in 1.8 to 2 Gy fractions). Although the study was not randomized, baseline patient characteristics and treatment variables did not differ significantly between both groups. Overall survival was similar in both groups, yet the group treated with radiotherapy quickly developed intra- and extrahepatic tumors outside the irradiation field resulting in a significantly shorter extrahepatic progression-free survival (Chung et al., 2006). The smaller study by Cheng et al. had similar findings (Chia-Hsien Cheng et al., 2001). A mechanism postulated is the irradiation-induced upregulation of VEGF and enhanced intra-tumor angiogenesis (Chung et al., 2006). In line with these findings, in 12 of 16 stage III rectal cancer patients, upregulation of VEGF was shown immunohistochemically on the resection specimen after neo-adjuvant radiochemotherapy (Nozue et al., 2001).

In a prospective randomized trial, patients with oro- and hypopharyngeal squamous cell carcinoma were either treated with surgery or with radiotherapy (5 x 4Gy) followed 3 days later by surgery. No difference in survival was seen, yet the group treated with radiotherapy had a higher incidence of distant metastases at time of death (Strong et al., 1978). Other non-randomized studies in head and neck cancer reported more distant metastases in the radiotherapy groups; yet selection bias could be the cause of this difference (Merino et al., 1977; Schantz & Peters, 1987).

These findings bring forth the hypothesis that the beneficial local effects of radiotherapy are counteracted by its systemic pro-invasive and pro-metastatic effect and therefore does not lead to an increased overall survival. The fact that the appearance of distant metastasis has been unchanged in the field of head and neck squamous cell carcinoma over the last decades, notwithstanding new radiotherapy and chemotherapy, supports this hypothesis (Duprez et al., 2017). The addition of molecules blocking this systemic pro-invasive and pro-metastatic effect could therefore entail a survival benefit, examples of these molecules are denoted in Table 2. The combination of radiotherapy with targeted molecules has been previously reviewed by Maier et al. (Maier et al., 2016).

Furthermore, it has been shown that radiotherapy and chemotherapy can alter the metastatic pattern as compared to no treatment. De La Monte et al. found that the distribution of metastases of small cell lung carcinoma was different after radiotherapy, chemotherapy or no therapy, but with similar number of total distant metastases and similar overall survival in all groups. These cancer treatments therefore might instigate a growth advantage to certain subpopulations of the tumor, which metastasize to specific sites (de la Monte et al., 1988).

Radiotherapy can also induce a local pro-invasive effect, as shown predominantly in breast cancer but also in nasopharyngeal and endometrial cancer, in which inflammatory skin recurrence, exactly demarcating the previously irradiated area, occurred (Tsujino et al., 2011; Meltzer et al., 1981; Marley & Marley, 1982). This phenomenon is rare and its incidence in breast cancer is estimated at 0.25%. The mechanism inducing it is not understood, yet radiotherapy could change the local microenvironment, facilitating tumor cells to migrate into this area (Tsujino et al., 2011).

In line with this, radiotherapy of prostate cancer and rectal cancer can increase MMP activity in the tumor and the local microenvironment (Angenete et al., 2009; Hovdenak et al., 2002; Kumar et al., 2002; Kumar et al., 2000). As stated above, these MMPs play a crucial role in invasion (Brown & Murray, 2015) and are correlated with cancer stage, metastasis and survival (Cho et al., 2005; Trudel et al., 2010).

Evidently, radiotherapy can also yield an anti-invasive and anti-metastatic effect. Post-mastectomy radiotherapy in high-risk breast cancer patients leads to a significantly lower probability of locoregional recurrences and distant metastases (Nielsen et al., 2006; Poortmans et al., 2015). Furthermore, several pre-clinical and clinical data indicate that radiotherapy can instigate a systemic anti-tumor effect in non-irradiated metastases, yet this effect is extremely rare and impossible to predict (Abuodeh et al., 2016; Formenti & Demaria, 2009). The underlying mechanism could be a radiotherapy-induced systemic anti-tumor immune response (Formenti & Demaria, 2009). The systemic anti-tumor effect of radiotherapy was described for the first time by Mole in 1953 and was termed the ‘abscopal effect’ (Mole, 1953). Recently, it has been more frequently reported, often when radiotherapy was administered in combination with checkpoint-inhibitors (Postow et al., 2012). Small prospective studies and retrospective studies have shown that the combination of checkpoint-inhibitors with radiotherapy could act synergistically, raising response rates and improving survival (Shaverdian et al., 2017; Twyman-Saint Victor et al., 2015; Hiniker et al., 2016; Williams et al., 2017; Tang et al., 2017; Sundahl et al., 2018). The underlying molecular mechanism could be the release of tumor antigen, triggered by radiotherapy-induced immunogenic cell death, and a subsequent systemic anti-tumor immune response, mediated by the checkpoint-inhibitor which reactivates the immune system by blocking the inhibitory signals on the immune cells (e.g. programmed cell death protein 1) (Formenti & Demaria, 2009). Several prospective clinical trials are currently investigating this combination treatment (Vacchelli et al., 2016; Sundahl et al., 2017; De Wolf et al., 2017).

## Conclusion

There is abundant evidence from in vivo and in vitro experiments that ionizing radiation, applied to cancer cells or host cells, to the tumor, the whole host or the putative site of tumor development may stimulate the metastatic process. Clinical observations do not directly confirm these data, leaving open the question whether or not radiotherapy enhances metastasis. The overall net outcome of radiotherapy is beneficial as it may reduce metastasis and prolong survival. This therapeutic benefit does not exclude a limitation of the benefit by negative effects of the irradiation on the cancer cells or on the host. The present review of literature suggests clinical trials to investigate correction of the pro-invasive and pro-metastatic activities of radiation by anti-inflammatory or other agents shown to be effective in preclinical settings.

## Abbreviations

Bcl-X_L_: B cell lymphoma-extra large
CAM: Chorioallontoic membrane
CCL: Chemokine ligand
c-MET: Tyrosine-protein kinase Met
CTC: Circulating tumor cells
CTLA4: Cytotoxic T-lymphocyte-associated protein 4
CYR61: Cysteine-rich angiogenic inducer 61
DTC: Disseminated tumor cells
EGFR: Epidermal growth factor receptor
EMT: Epithelial mesenchymal transition
FAK: Focal adhesion kinase
Gadd45a: Growth arrest and DNA damage inducible protein alpha
G-CSF: Granulocyte-colony stimulating factor
GSK-3β: Glycogen synthase kinase-3β
GTP: Guanosine triphosphate
Gy: Gray
HGF: Hepatocyte growth factor
HIF: Hypoxia inducible factor
HUVEC: Human umbilical vein endothelial cells
ILK: Integrin-linked kinase
IκB: Inhibitor of nuclear factor of kappa-light-chain-enhancer of activated B cell
LAP-β1: Latency-associated peptide of TGFβ
MIF: Migration inhibitory factor
MMP: Matrix metalloproteinase
MSC: Mesenchymal stromal cell
MT1-MMP: Membrane-bound matrix metalloproteinase-1
PI3K: Phosphoinositide 3-kinase
PINCH1: Particularly interesting new cysteine-histidine-rich 1
PTEN: Phosphatase and tensin homolog
ROS: Reactive oxygen species
Snail: Zinc finger protein SNAI1
STAT3: Signal transducer and activator of transcription factor 3
TBI: Total body irradiation
TBK-1: Tank-binding kinase-1
TCF: T-cell factor
TIMP: Tissue inhibitors of matrix metalloproteinase
Trex1: Three prime repair exonuclease 1
uPA: Urokinase-type plasminogen activator
VCAM: Vascular cell adhesion molecule
VEGF: Vascular endothelial growth factor
ZEB1: Zinc finger E-box-binding homeobo× 1
